# A systematic review and meta-analysis of randomised controlled trials on surgical treatments for ingrown toenails part I: recurrence and relief of symptoms

**DOI:** 10.1186/s13047-023-00631-1

**Published:** 2023-06-10

**Authors:** Victoria Exley, Katherine Jones, Grace O’Carroll, Judith Watson, Michael Backhouse

**Affiliations:** 1 York Trials Unit, Department of Health Sciences, University of York, York, UK; 2 Warwick Clinical Trials Unit, Warwick Medical School, University of Warwick, Coventry, CV4 7AL UK

**Keywords:** Ingrown nail, Ingrown nails, Nail, Malformed, Onychocryptosis, Nail surgery, Nail avulsion, Review, Systematic

## Abstract

**Background:**

Ingrown toenails are a common nail pathology. When conservative treatments are ineffective, a surgical approach is often utilised. Despite recent narrative reviews, there is a need for an up-to-date and rigorous systematic review of surgical methods for treating ingrown toenails.

**Methods:**

Five databases (MEDLINE, Embase, CINAHL, Web of Science and CENTRAL) and two registers (Clinicaltrials.gov and ISRCTN) were searched to January 2022 for randomised trials evaluating the effects of a surgical intervention(s) for ingrown toenails with a follow-up of at least 1 month. Two independent reviewers screened records, extracted data, assessed risk of bias and certainty of evidence.

**Results:**

Of 3,928 records identified, 36 (3,756 participants; 62.7% males) surgical interventions were included in the systematic review and 31 studies in the meta-analysis. There was very low quality evidence that using phenol with nail avulsion vs nail avulsion without phenol reduces the risk of recurrence (risk ratio [RR] 0.13 [95% CI 0.06 to 0.27], *p* < 0.001). No favourable effect was observed between chemical or surgical vs conservative management (0.55 [0.19 to 1.61], *p* = 0.280; 0.72 [0.33 to 1.56],* p* = 0.410), chemical or surgical vs other (e.g., CO_2_ laser, electrocautery) (1.61 [0.88 to 2.95], *p* = 0.120; 0.58 [0.25 to 1.37], *p* = 0.220), chemical vs surgical (0.75 [0.46 to 1.21], *p* = 0.230), surgical vs surgical (0.42 [0.21 to 0.85]), chemical vs chemical (0.19 [0.01 to 3.80], *p* = 0.280), surgical vs surgical + chemical (3.68 [0.20 to 67.35], *p* = 0.380), chemical vs surgical + chemical (1.92 [0.06 to 62.30], *p* = 0.710), local anaesthetic vs local anaesthetic + adrenaline (1.03 [0.22 to 4.86], *p* = 0.970), chemical timings 30 s vs 60 s (2.00 [0.19 to 21.41]) or antibiotics vs no antibiotics (0.54 [0.12 to 2.52], *p* = 0.430). Central toenail resection was the only procedure to significantly relieve symptoms (*p* = 0.001) but data were only available up to 8 weeks post-surgery.

**Conclusion:**

Despite the high number of publications, the quality of research was poor and the conclusions that can be inferred from existing trials is limited. Phenolisation of the nail matrix appears to reduce the risk of recurrence following nail ablation, and with less certainty 1 min appears to be the optimum time for application. Despite this being a widely performed procedure there remains a lack of good quality evidence to guide practice.

**Supplementary Information:**

The online version contains supplementary material available at 10.1186/s13047-023-00631-1.

## Introduction

An ingrown toenail, or onychocryptosis, is a common problem that occurs when the nail plate punctures the periungual skin causing substantial pain, inflammation, discomfort and increased risk of infection if left untreated [1]. Most cases occur in the hallux and typically present in teenagers and young adults, although may present at any age [2, 3]. Several factors have been proposed as contributory to the occurrence or worsening of ingrown toenails with varying degrees of evidence. These include poor nail cutting technique, hyperhidrosis, ill-fitting footwear, nail deformity, trauma, obesity, and peripheral oedema [4, 5].

Multiple semi-quantitative classification systems have been developed to classify ingrown toenails. Most focus on the severity of ingrown toenails, and generally have three stages: mild (stage I), moderate (stage II) and severe (stage III) [6–8]. Although, more recently an alternative approach has been suggested that focusses on the shape of the nail plate and aetiology of the pathology [8]. The performance of these classification systems has not been evaluated but have been proposed as a basis upon which to base treatment decisions.

Conservative approaches in the form of appropriate nail cutting and spicule removal, soaking in warm water, guttering, and orthonyxia (nail bracing) have all been advocated in the literature for use in mild to moderate stages (stage I and II) with varying success rates and quality of evidence [5, 8]. However, when conservative treatment fails, where there is nail deformity, or in more severe cases (stage II and III), a surgical approach is often recommended aiming to remove the problem part of the nail and destroy the underlying matrix to avoid recurrence [8–10].

Multiple surgical interventions have previously been described with most including either partial or total avulsion of the nail plate, that is often combined with ablation of the nail matrix to stop regrowth. Nail surgery is performed by a range of health professionals including GPs (general practitioners), orthopaedic surgeons, dermatologists, and podiatrists. Indeed nail surgery forms a substantial part of the workload of podiatrists, having been identified as the tenth most commonly performed procedure performed by the profession [11]. Whilst there is little published data to describe how this common nail pathology is treated in practice, it is clear that a large number of small studies have been published on the topic. Systematically searching for and reviewing these studies, pooling estimates of effectiveness, and providing recommendations for practice and future research is essential to enable evidence-based practice.

A Cochrane review published 10 years ago suggested that use of phenol reduced the likelihood of recurrence but did not differentiate between regrowth of the nail plate (which may be asymptomatic) with recurrence of symptoms [4]. The authors also found that there was insufficient evidence to make recommendations on whether more radical surgery was more effective in cases of more severe disease, or how key patient reported outcomes such as relief of symptoms, patient satisfaction, and post-operative pain were affected by nail surgery. More recent narrative reviews have been written [8, 12], and a systematic review specific to the use of phenol used a very limited search strategy, and did not adhere to key methodological principles such as prospective registration of their review [13]. Accordingly, there is a need for an up-to-date and rigorous systematic review of surgical methods for treating ingrown toenails. The aim of this study, therefore, was to systematically search and synthesise the literature relating to the effectiveness/efficacy of surgical methods for treating ingrown toenails.

## Methods

The Cochrane Handbook for Systematic Reviews of Interventions [14] was used to guide the conduct of this review. The review was reported in accordance with the Preferred Reporting Items for Systematic Reviews and Meta-Analyses (PRISMA) [15], This review was prospectively registered at https://www.crd.york.ac.uk/prospero/ [CRD42021251938].

### Eligibility criteria

Randomised controlled trials (RCTs) that evaluated the effects of a surgical intervention(s) for ingrown toenails with a follow-up period of at least 1 month were included. Trials comparing one form of surgery with another form of surgery, or a non-surgical intervention, or no intervention were included. Unpublished trials and conference abstracts were only included if the methodological descriptions were adequate to determine eligibility. Where such information was missing from the abstract, it was sought through direct contact with the author. There were no restrictions on the setting, age, or gender of participants. Studies were restricted to English, pertaining to human participants, and must have reported one of the following outcomes for inclusion. Relief of symptoms, and symptomatic regrowth (nail spicules/nail spikes) were considered co-primary outcomes in advance of conducting the searches. Healing time, postoperative complications (e.g., infection and haemorrhage), pain of operation, postoperative pain (duration and intensity) and participant satisfaction were defined as secondary outcomes. After completing searches, and screening it became clear that the majority of papers did not differentiate symptomatic/asymptomatic regrowth of the nail plate, but instead frequently conflated these and considered ‘recurrence’. This was therefore adopted as a co-primary outcome rather than ‘symptomatic regrowth’.

### Search strategy

Electronic databases were searched from inception to January 2022: MEDLINE (Ovid), EMBASE (Ovid), CINAHL, Web of Science and Cochrane Central Register of Controlled Trials (CENTRAL). The search strategy (Supplementary File 1) was conducted using Medical Subject Heading (MeSH), truncation and Boolean operators. Other relevant completed and ongoing studies were also sought through screening of clinicaltrials.gov, the International Clinical Trials Registry [ISRCTN] and forward and backwards citations of included studies.

All searches were carried out by the same author and search results generated by the electronic databases were exported to Rayyan, where duplicates were removed. Abstract, titles and full text screening were conducted independently by two review authors, who recorded reasons for exclusion. Discrepancies were discussed with a third author and resolved by consensus. Review authors were not blinded to the author, institution, or the publication source of the study.

### Data extraction

A modified Cochrane data extraction form was piloted and then used to extract and record information. Data extracted included: (a) general information such as author(s), title, journal and study funding; (b) trial characteristics such as study aim and objectives, study design, unit of allocation and ethical approvals; (c) participant characteristics such as setting, inclusion/exclusion criteria, sample size (number of participants and nail folds), age, gender, baseline imbalances, severity of ingrown toenails; (d) intervention and comparison group(s); (e) outcome measures including as time points, unit of measurement, outcome definition, data at baseline/follow-up and statistical methods.

Two review authors independently extracted data from the included studies, with disagreements resolved through consensus of a third review author. Where data were missing or unclear, the corresponding author(s) was contacted via email and relevant information requested. If after initial request no response was forthcoming, at least one further email was sent.

### Data synthesis and analysis

Meta-analyses were conducted using Review Manager (RevMan V5.3 Cochrane Collaboration, Oxford, UK). The RevMan programme is designed to allow independent data entry by different reviewers. This ensures blinded data entry and highlights any discrepancies in values entered by reviewers. Any discrepancies between entered values was rechecked and discussed. For continuous data, pooled results were expressed as mean differences (MD), 95% confidence intervals (CI) and *p* values, with <0.05 considered significant. For dichotomous outcomes, pooled results were expressed as risk ratios (RR) and corresponding 95% CI, generated by RevMan. Where studies used a different assessment tool to measure the same construct, the standardised mean difference (SMD) and corresponding 95% CI was calculated. Where studies had multiple assessment time points, data were extracted for the final follow-up time point from randomisation.

Statistical heterogeneity and consistency were determined by interpreting the I^2^ statistic, and the following thresholds were identified a priori: 0–40% may not be important, 30–60% may represent moderate heterogeneity, 50–90% may represent substantial heterogeneity, 75–100% considerable heterogeneity [14]. If statistical heterogeneity was noted (I^2^ > 40%) for a particular treatment comparison, a random-effects model was used for analysis to account for expected heterogeneity between studies. Where there was no or little evidence of statistical heterogeneity, a fixed-effects model was used. The fixed-effect model assumes all studies are measuring the same treatment effect and that all differences between studies are due to random (sampling) error. The Mantel-Haenszel methods [16] is the default fixed-effect method implemented in Revman. The random-effects model assumes the treatment effect varies between the studies. This model estimates the mean of the distribution of effects and is weighted for both within-study and between-study variation. This approach uses the variance within each study and adds a second measure known as Tau^2^. Sensitivity analysis was performed if a substantial heterogeneity (I^2^ > 75%) was detected. Where data aggregation was not possible due to methodological heterogeneity, these results were summarised narratively. Potential publication bias for each outcome with more than ten studies was evaluated by visual inspection of funnel plots [17].

### Certainty of evidence

The Grades of Research, Assessment, Development and Evaluation (GRADE) approach was used independently by two review authors to analyse the certainty of evidence against six domains: risk of bias/ certainty, indirectness of evidence, heterogeneity or inconsistency of effect, imprecision and publication bias [18, 19]. Disagreements were resolved by a third author.

### Risk of bias

The Cochrane Risk of Bias tool (RoB 2.0) was assessed independently by two review authors to determine the validity and methodological rigor [18]. Discussion between the two review authors was utilised to resolve any discrepancies, with any disagreements resolved by a third author. Included studies were assessed on the randomisation process, deviations from the intended interventions, missing outcome data, measurement of the outcome and selection of the reported result, with each domain judged as ‘low’, ‘some concerns’, or ‘high’ risk. The overall RoB judgement was derived from the highest classified domain.

In this paper we present analysis for our primary outcomes and secondary outcomes are presented in a subsequent paper.

## Results

### Search results

**Fig. 1:**
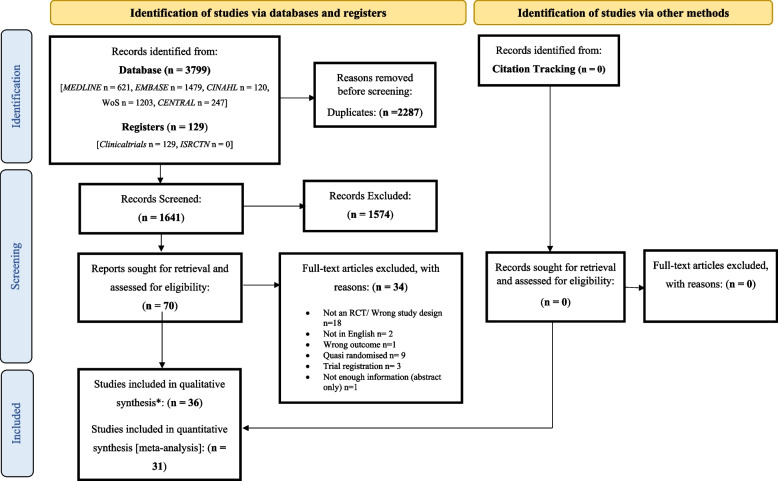
PRISMA flow diagram of literature search and study selection phases. n, number; WoS, Web Of Science; CENTRAL, Cochrane Central Register of Controlled Trials; WHO ICTRP, World Health Organisation International Clinical Trials Registry Platform; ISRCTN, International Standard Randomised Controlled Trial Number Registry

A total of 3,928 records were identified, with 1,641 remaining after de-duplication, 70 studies were retrieved and examined for full-text screening leaving 36 studies eligible for inclusion (Fig. 1 and Supplementary Table 1). Of these, 31 were included in the meta-analysis for recurrence and 5 were reported narratively. In accordance with Cochrane Handbook guidance [Section 23.3], the study by Tatlican and colleagues [20] was not included in the meta-analysis for the following reasons: 1) groups could not be combined due to the similarity in groups, comparison of the same chemical at different application timepoints, 2) all groups were relevant therefore it was not possible to include only relevant groups, and 3) as all groups were being compared there was no single comparator to spilt the ‘shared’ group into two or more groups. Other reasons for exclusion from the meta-analysis included not assessing the outcome measure [21], heterogeneity in terminology reporting [22–24] and three trial arms [20].

In addition, synthesis of evidence for relief of symptoms was reported narratively due to the small number of studies reporting this outcome.

### Study characteristics

Characteristics of included studies are summarised in Table 1. Of the 36 RCTs, published between 1979 to 2021, studies were conducted across 14 countries with Turkey being the most frequent (16.6%). Included studies comprised of 3,756 participants, with a sample size ranging from 10 to 125 per study. Five studies [22–26] did not report gender. Of the remaining 31 studies, 62.7% of participants were male.

**Table 1:** Characteristics of included studies

**Author (Year)**	**Study Design (Country)**	**Follow-Up**^**a**^	**Participants**	**Interventions**	**Outcome Measures**
Ahsan (2019) [27]	RCT (Pakistan)	3 months	100 participants; Mean age 26.31 + 8.74; 45/46 M/F; Severity not reported	A: Chemical matrixectomy with phenol (*n* = 50)	1. Healing time at 48 h, week 1, 4 and 12 up to 3 months. 2. Wound infection and discharge 3. Pain (no pain, mild, moderate, severe) 4. Recurrence up to 3 months
				B: Chemical matrixectomy with trichloroacetic acid (*n* = 50)	
Akkus (2018) [28]	RCT (Turkey)	12 months	60 participants (75 procedures); Mean age 30 – 31.7; 27/23 M/F; Stage I (*n* = 32), Stage II (*n* = 19), Stage III (*n* = 24)	A: Chemical matrixectomy with NaOH (*n* = 30)	1. Recurrences at 12 months 2. Healing time assessed at day 3, Week 1, Month 1, 6, and 12 3. Pain (pain severity- mild, moderate or severe) 4. Operation time 5. Post-operative drainage 6. QOL (Dermatologic Life Quality Index)
				B: Wedge resection (*n* = 30)	
AlGhamdi (2014) [29]	RCT (Saudi Arabia)	6 months	53 participants; Mean age 47.7 + 1.3 (range 10–60 years); 46/7 M/F; Stage II and III	A: Lateral nail avulsion with phenol (*n* = 30)	1. Symptomatic regrowth 1, 3 and 6 months 2. Healing time 1, 3 and 6 months 3. Significant complications including infection 4. Pain at baseline, 1, 3 and 6 months 5. Participant satisfaction 6. Drainage 7. Shoe-wear discomfort 8. Overall success rate
				B: Nail tube splinting (*n* = 23)	
Altinyazar (2010) [30]	RCT (Turkey)	18 months	44 participants; Mean 28.5–32.8; 21/23 M/F; All stage III	A: Plain lidocaine (*n* = 22)	1. Recurrence at 18 months 2. Healing time 3. Pain 4. Drainage
				B: Lidocaine with epinephrine (*n* = 22)	
Alvarez-Jimenez (2011) [31]	RCT; Double- Blinded (Spain)	12 months	51 participants (152 procedures); Mean age 34.05 + 19.23; 18/33 M/F; Stage I or IIa	A: Phenol and curettage (*n* = 73 nail folds)	1. Recurrence at 12 months 2. Healing time over 1 months (digital photo) 3. Infection 4. Bleeding over 48 h 5. Pain 2 days post-surgery (VAS)
				B: Phenol (*n* = 79 nail folds)	
Anderson (1990) [32]	RCT (UK)	12 months	31 participants; Age range 15–73 years); 19/11 M/F; Severity not reported	A: Nail bed excision (*n* = 17)	1. Recurrence at 12 months 2. Post operative infection at 2 weeks 3. Participant satisfaction at 2 weeks and 12 months 4. Previous operations and relation to recurrence
				B: Combination of nail bed phenolisation and excision (*n* = 14)	
Andre (2018) [33]	RCT; Double- Blinded (France)	4 months	84 participants (96 toenails); Age range 14–88 years; 41/40 M/F; Severity not reported	A: Nail avulsion and phenol (*n* = 46 toenails)	1. Recurrence at 4 months 2. Inflammation at day 2, week 2, 4 and month 4 3. Pain at 34 days post-surgery (VAS) 4. Presence of oozing 5. Postoperative morbidity
				B: Nails avulsion and trichloroacetic acid (n = 50 toenials)	
Awad (2020) [34]	RCT (Saudi Arabia)	6 months	200 participants; Mean age 26.45 (range 16–52 years); 136/64 M/F; Stage I (*n* = 66), Stage II (*n* = 38), Stage III (*n* = 96)	A: Partial nail matrixectomy with electrocautery (*n* = 100)	1. Recurrence at 1 and 6 months 2. Healing time assessed at 3 and 7 days, 1 and 6 months 3. Infection assessed at 3 and 7 days 4. Pain assessed at day 3 and 7 (VAS) 5. Participant satisfaction at 1 and 6 months
				B: Partial nail matrixectomy (*n* = 100)	
Bos (2007) [35]	RCT (Netherlands)	12 months	123 participants; Age range 9–73; 72/45 M/F; Severity not reported	A: Partial avulsion with excision of the matrix (*n* = 38)	1. Recurrence at 1, 6 and 12 months 2. Infection at 2 days, 1 week and 1 month
				B: Partial avulsion of the matrix and application of antibiotic (*n* = 22)	
				C: Partial avulsion with application of phenol (*n* = 37)	
				D: Partial avulsion with application of phenol and antibiotic (*n* = 26)	
Ceren (2013) [36]	RCT (Turkey)	6 months	120 toenails; Age range (11–65 years); 59/48 M/F; Stage I (*n* = 31), Stage II (*n* = 17), Stage III (*n* = 72)	A: Partial nail extraction with phenol matrixectomy (*n* = 63 toenails)	1. Recurrence at 6 months 2. Haemorrhage or discharge at 6 months 3. Pain at 6 months 4. Cosmetic satisfaction at 6 months 5. Time to recovery
				B: Partial nail elevation and flexible tube (57 toenails)	
Cordoba-Fernandez (2015) [37]	RCT (Spain)	12 months	44 participants (10 toes); Mean age 26.28 + 15.82 (range 10–64 years); 21/23 M/F; Severity not reported	A: Segmental phenolisation matrixectomy with anaesthetic digital block with epinephrine (*n* = 34 toes)	1. Recurrence 2. Bleeding 3. Pain at 1-, 2- and 3-days post-op (VAS) 4. Duration of anaesthetic effect
				B: Segmental phenolisation matrixectomy with anaesthetic digital block without epinephrine (36 toes)	
Gem (1990) Study 1[22]	Prospective RCT (UK)	18 months	219 participants; age, gender and severity not reported	A: Chemical ablation with 3-min application of 80% phenol (*n* = 109)	1. Recurrence at 18 months 2. Time to become pain free 3. Relief of symptoms 4. Healing time
				B: Chemical ablation with 2-min application of 10% sodium hydroxide (*n* = 110)	
Gem (1990) Study 2 [23]	Prospective RCT (UK)	18 months	203 participants; age, gender and severity not reported	A: Chemical ablation with 2-min application of 10% sodium hydroxide (*n* = 110)	1. Recurrence at 18 months 2. Time to become pain free 3. Relief of symptoms 4. Healing time
				B: Chemical ablation with 1-min application of 10% sodium hydroxide (*n* = 93)	
Gerritsma-Bleeker (2002) [38]	RCT (Netherlands)	12 months	60 participants (63 procedures); Mean age 22.7- 24.4; 30/33 M/F; Severity not reported	A: Partial nail extraction with phenolisation (*n* = 31)	1. Relief of symptoms at 1, 3 and 12 months (VAS) 2. Recurrence at 1, 3 and 12 months 3. Pain at 2 days, 8 days, 1 month, 3 months, 12 months (VAS) 4. Participant satisfaction with scar and cosmetic result at 1, 3 and 12 months (VAS) 5. Erythema & purulent exudates 6. Morbidity 7. Time to complete recovery
				B: Partial nail extraction with matrix excision (*n* = 34)	
Greig (1991) [39]	RCT (Scotland)	12 months	163 participants (204 procedures); Age range 12–77 years; 113/50 M/F; Severity not reported	A: Total avulsion (*n* = 81 nail edges)	1. Recurrences at 12 months 2. Postoperative infection after 2 weeks 3. Participant satisfaction at 12 months
				B: Nail edge excision (*n* = 56 nail edges)	
				C: Nail edge excision and phenolisation (*n* = 67 nail edges)	
Habeeb (2020) [40]	RCT (Egypt)	6 months	100 participants; Age range 10–35 years; 78/22 M/F; Severity not reported	A: Central toenail resection (*n* = 50)	1. Relief of symptoms at 4 and 8 weeks 2. Recurrences at 6 months 3. Pain at 1,2,3 and 4 days post-op 4. Duration of technique
				B: Wedge toenail resection (*n* = 50)	
Hamid (2021) [25]	RCT (Pakistan)	6 months	100 participants; Mean age 19.7–20.2; gender and severity note reported	A: Partial nail avulsion and matrixectomy with phenol (*n* = 50)	1. Recurrence at 3 and 6 months 2. Serous and Purulent discharge at 7th and 14th days and at week 4 and 6 3. Pain
				B: Partial nail avulsion and matrixectomy with electrocautery (*n* = 50)	
Issa (1998) [41]	RCT (Ireland)	6 months	140 participants (170 procedures); Mean age 21 (range 9–54 years); 65/22 M/F; Severity not reported	A: Wedge resection and segmental phenolisation combination treatment (*n* = 62)	1. Recurrence at 6 months 2. Pain duration at 1–6 h, 6–12 h, 12–24 h, > 24 h post-op 3. Pain intensity using the linear pain analogue scale
				B: Wedge resection (*n* = 55)	
				C: Segmental phenolisation (*n* = 53)	
Kavoussi (2020) [42]	RCT (Iran)	24 months	127 participants; Mean age 28.2–28.9; 68/59 M/F; Stage I (*n* = 7), Stage IIa (*n* = 40), Stage IIb (*n* = 51), Stage III (*n* = 21), Stage IV (*n* = 8)	A: Partial Nail Matrixectomy using CO_2_ laser (*n* = 62)	1. Spicule formation 2. Healing time 3. Infection 4. Pain 5. Cosmetic outcome 6. Time to return to daily activity 7. Time to return to work
				B: Lateral Nail Fold Excision (LNFE) (*n* = 65)	
Khan (2014) [43]	RCT (Pakinstan)	6 months	100 participants; Mean age 18 (range 14–45 years); M:F ratio = 2.3:1; Severity not reported	A: Partial Nail Avulsion + Phenol (*n* = 50)	1. Recurrence or spike formation at 1 and 6 months 2. Infection at 3^rd^ and 7^th^ day post-op 3. Pain at 3^rd^ and 7^th^ day post-op (VAS) 4. Mortality
				B: Partial Nail Avulsion alone (*n* = 50)	
Kim (2015) [44]	RCT (Korea)	6 months	61 participants; Mean age 20.0 – 20.3; All male; Grade 2 or 3	A: Curettage (*n* = 32)	1. Recurrences at 6 months 2. Infection at 3–5 days post-op
				B: Electrocautery (*n* = 29)	
Korkmaz (2013) [45]	RCT (Turkey)	Mean 3.2 ± 1.2 years	39 participants; Mean age 16.1–17.0; 28/11 M/F; Stage 2 (*n* = 13) or 3 (*n* = 26)	A: Partial matrix excision (*n* = 17)	1. Recurrence 2. Infection 3. Pain (Duration of analgesic usage) 4. Return to work time
		Mean 2.1 ± 0.9 years		B: Segmental phenolisation (*n* = 22)	
Kruijff (2008) [46]	RCT (Netherlands)	12 months	105 participants (109 nails procedures); Mean age 25.3 + 15.2; 73/36 M/F; Severity not reported	A: Partial nail extraction with partial matrix excision (*n* = 58)	1. Recurrence at 12 months 2. Redness, exudate and post-operative bleeding at 1 week 3. Pain at 4, 12 and 26 weeks (VAS) 4. Participant satisfaction at 4 and 26 weeks (VAS) 5. Time to complete recovery 6. Relief of symptoms (VAS)
				B: Orthonyxia (*n* = 51)	
Leahy (1990) [47]	RCT (Ireland)	30 months	66 participants; Mean age 24 years; 48% females; Severity not reported	A: Chemical ablation (phenol) (*n* = 32)	1. Spicules or spikes regrowth at 3 months and between 16 and 30 months 2. Infection at 1 week, 3 months, and between 16 and 30 months 3. Haemorrhage at 1 week, 3 months, and between 16 and 30 months 4. Cosmetic outcome between 16 and 30 months 5. Postoperative pain (absence of pain relief) at 1 week, 3 months and between 16 and 30 months 6. Participant satisfaction at 16 and 30 months
				B: Surgical ablation (*n* = 34)	
Misiak (2014) [48]	RCT (Poland)	3 months	60 participants; Mean age 41.4 + 9.95 (range 26–64 years); 32/28 M/F; Grade 3 and 4	A: Partial nail extraction + phenolisation (*n* = 30)	1. Recurrence at 1, 2 and 3 months 2. Healing time at 7 days, 14 days, 1, 2 and 3 months
				B: Partial nail extraction + electrocautery (*n* = 30)	
Morkane (1984) [49]	RCT (New Zealand)	14 months	103 participants (107 procedures); Mean age 24.6 – 28.5; M/F ratio 3.5:1; Severity not reported	A: Segmental or angular phenolisation (*n* = 54)	1. Regrowth at mean follow up time of 14 months 2. Pain measured 1 week post-operatively (linear analogue scale)
				B: Wedge excision (*n* = 53)	
Muriel-Sánchez (2020) [50]	RCT (Spain)	6 months	34 participants (112 procedures); Mean age 34 + 18.3; 12/22 M/F; Stage I or IIa	A: Chemical matrixectomy with phenol (*n* = 10)	1. Recurrence at 6 months 2. Healing time 3. Post-surgical bleeding at first dressing 4. Infection 5. Pain at 24, 48, 72 h (VAS) 6. Post-surgical inflammation
				B: “Aesthetic reconstruction” (describes partial nail ablation with wedge excision of matrix) (*n* = 24)	
Muriel-Sánchez (2021) [51]	RCT (Spain)	6 months	27 participants (108 procedures); Mean age 36 + 10.7 years; 8/19 M/F; Stages I or IIa	A: Partial nail avulsion with 30 s application of phenol (*n* = 27 halluces [54 nail folds])	1. Recurrence at a minimum of 6 months 2. Healing time 3. Inflammation, bleeding & infection at 72 h then twice weekly until healed 4. Pain at 1, 2 and 3 days post-op (VAS)
				B: Partial nail avulsion with 60 s application of phenol (*n* = 27 halluces [54 nail folds])	
Peyvandi (2011) [52]	Prospective RCT (Iran)	6 months	100 participants; Mean age 27.8 (range 12–47 years); 54/46 M/F; Severity not reported	A: Winograd method (*n* = 50)	1. Recurrence at 1 week, 1 and 6 months 2. Infection at 1 week, 1 and 6 months 3. Postoperative workday loss 4. Surgery duration 5. Participant satisfaction
				B: Sleeve (gutter) method (n = 50)	
Reyzelman (2000) [21]	RCT (US)	Until healed	154 participants; Mean age 20.7 + 8.6 (range 10–60 years); 91/63 M/F; Severity not reported	A: 1 week course of oral antibiotics and simultaneous phenol matrixectomy (*n* = 53)	1. Healing time assessed at 3–4 days then weekly until healing occurred 2. Infection assessed at 3–4 days then weekly until healing occurred
				B: 1 week course of oral antibiotics and phenol matrixectomy 1 week later (*n* = 51)	
				C: Phenol matrixectomy without antibiotic therapy (*n* = 50)	
Shaath (2005) [53]	RCT (UK)	12 months	83 participants; Mean age 37.2 – 39.4 years; 53/30 M/F; Severity not reported	A: Zadik’s procedure (*n* = 38)	1. Symptomatic regrowth 2. Pain at 3 & 6 weeks (VAS) 3. Return to shoe wear 4. Return to normal activity 5. Number of dressings
				B: Chemical ablation with Sodium Hydroxide (*n* = 45)	
Tatlican (2009) [20]	RCT (Turkey)	24 months	110 participants (148 procedures); Mean age 31.6–32.7 years. 54/56 M/F; Grade 2 (*n* = 65) or 3 (*n* = 83) ingrowing nail	A: Partial nail avulsion with 1 min phenol cauterisation (*n* = 37)	1. Recurrence at 6-month intervals for 24 months 2. Healing time. Examined on alternate days until healing achieved 3. Pain at 2^nd^, 10^th^, 16^th^, 24^th^ & 30^th^ days of follow up 4. Drainage and tissue damage at 2^nd^, 10^th^, 16^th^, 24^th^ & 30^th^ days of follow up
				B: Partial nail avulsion with 2-min phenol cauterisation (*n* = 36)	
				C: Partial nail avulsion with 3-min phenol cauterisation (*n* = 37)	
Uygur (2016) [54]	Prospective RCT (Turkey)	6 months	128 participants; Mean age 22.8 (range 12–48 years); 83/41 M/F; Heifetz stage I (*n* = 28, 23%), Stage I (*n* = 73, 58%), Stage III (*n* = 23, 18%)	A: Winograd procedure and new suturing technique (n = 64)	1. Recurrence at 2 weeks, 1 and 6 months 2. Satisfaction at suture removal, 1 and 6 months 3. Time elapsed before shoes could be worn at 2 weeks, 1 and 6 months 4. Return to work/school
				B: Winograd procedure and traditional suturing technique (*n* = 64)	
Van der Ham (1990) [55]	RCT (Netherlands)	14 months	249 participants; Age range 3–97 years; 158/ 91 M/F; Severity not reported	A: Wedge excision (*n* = 124)	1. Analgesic required after 7 days and weekly until healed for 14 months 2. Sick leave 3. Recurrence for 14 months 4. Re-operation 5. Nail Spikes 6. Healing time 7. Time required off work
				B: Segmental phenol cauterisation (*n* = 125)	
Varma (1983) [26]	RCT (UK)	6 months	67 participants; Age, gender and severity not reported	A: Surgical wedge excision (*n* = 35)	1. Symptomatic recurrence at 3 and 6 months 2. Healing time at 1 week, 1 and 3 months
				B: Phenol wedge cauterisation (*n* = 28)	
Wallace (1979) [24]	Prospective RCT (UK)	15 months	68 participants; Age range 10 to 73 years; gender and severity not reported	A: Gutter treatment (*n* = 32)	1. Number of successes/Number of failures 2. Number having no further operation/ Number undergoing reoperation 3. Pain after 1 day
				B: Wedge resection (*n* = 36)	

*M/F* Male/Female, *VAS* Visual Analogue Scale, *RCT* Randomised Controlled Trial, *QOL* Quality of Life, *NaOH* Sodium Hydroxide

^a^Final follow-up point

### Interventions

Of the 36 included studies, 5 compared a conservative intervention: orthonyxia [46], nail tube splinting [29], nail elevation and flexible tubing [36], or gutter method [24, 52] to either chemical or surgical matrixectomy. Eleven studies [26, 28, 35, 38, 41, 45, 47, 49, 50, 53, 55] compared chemical matrixectomy to surgical matrixectomy, using various techniques. Two studies [39, 43] compared chemical matrixectomy to avulsion only, and two studies [31, 41] compared chemical matrixectomy to a combination of surgical and chemical matrixectomy.

One study compared a surgical intervention to another surgical intervention [40], two added the use of phenol to a surgical intervention [32, 41]. Three studies compared the same surgical intervention, one adding phenol alongside partial nail avulsion [43], another compared nail avulsion with phenol or trichloroacetic acid [33] and one introduced a new suturing technique alongside a Winograd procedure [54]. One study compared chemical matrixectomy with phenol or trichloroacetic acid [27]. Four studies compared the same surgical intervention and introduced a chemical matrixectomy at different application timings [20, 22, 23, 51].

Five compared an alternative intervention: CO_2_ laser [42] or electrocautery [25, 34, 44, 48] to either chemical or surgical matrixectomy. There were 2 studies [30, 37] comparing local anaesthetics (4 mL solution of 2% mepivacaine; 2% plain lidocaine, respectively), with or without epinephrine and 2 studies [21, 35] looked at pre- and postoperative use of antibiotics following a surgical intervention.

#### Recurrence

Recurrence was reported in all but one study [21]. The definition of recurrence varied between studies (Table 2) and two studies were unclear and reported the ‘number of successes/number of failures’ [24] and ‘number symptom-free’ [22, 23], respectively. Therefore, these studies were reported narratively. Follow-up ranged from 1 to 24 months.

**Table 2:** Recurrence definitions

Ahsan (2019) [27]	No definition provided
Akkus (2018) [28]	No definition provided
AlGhamdi (2014) [29]	No definition provided
Altinyazar (2010) [30]	Recurrence was defined as occurrence of any clinical sign of regrowth of the treated nail edge, such as pain, discomfort, erythema, or drainage. Spicule formation, which shows the inadequate destruction of the germinal matrix, was also accepted as recurrence
Alvarez-Jimenez (2011) [31]	Recurrence rate was evaluated as growth of the released nail (or of a piece of the released nail) even though that recurrent nail might be asymptomatic
Anderson (1990) [32]	Recurrence was defined as any evidence of nail growth
Andre (2018) [33]	Recurrence was defined as the presence of a nail spicule or any sign of ingrowing nail
Awad (2020) [34]	No definition provided
Bos (2007) [35]	No definition applied at the study start; applied definition to see the impact on study results “If regrowth or spike formation at the site of the removed part of the nail was also considered as recurrence, together with recurrence of IGTN, the effect of antibiotics was not significant (*P* = 0·876) and phenolization remained significantly better than matrix excision (*P* < 0·001). The increase in number of recurrences when this definition was applied was mainly due to the significantly higher chance of nail regrowth when matrix excision was used (*P* = 0·019)
Ceren (2013) [36]	No definition provided
Cordoba-Fernandez (2015) [37]	Recurrence rate–was considered present when there was symptomatic regrowth (including nail spicules/inclusion cysts) or asymptomatic nail spikes after a minimum post-operative follow-up of 1 year
Gem (1990) a [22]	Unclear in their reporting of recurrence recording the ‘number symptom-free’
Gem (1990) b [23]	Unclear in their reporting of recurrence recording the ‘number symptom-free’
Gerritsma-Bleeker (2002) [38]	Recurrence was defined as evidence of ingrowth of the nail edge or spicule formation
Greig (1991) [39]	Recurrence was defined as evidence of ingrowth of the nail edge or spicule formation
Habeeb (2020) [40]	No definition provided
Hamid (2021) [25]	No definition provided
Issa (1998) [41]	Recurrence was defined by the presence of nail growth on the affected side, whether or not symptomatic, i.e. an asymptomatic nail spike was considered a recurrence
Kavoussi (2020) [42]	No definition provided
Khan (2014) [43]	No definition provided
Kim (2015) [44]	No definition provided
Korkmaz (2013) [45]	No definition provided
Kruijff (2008) [46]	Recurrence was defined as evidence of ingrowth of the nail edge or spicule formation
Leahy (1990) [47]	Number of spicules or spiked regrowth’s of nail occurring at the nail bed edge, remote from the main nail
Misiak (2014) [48]	No definition provided
Morkane (1984) [49]	Number of nail spikes out of total procedures
Muriel-Sánchez (2020) [50]	To measure recurrence, a relapse of clinical reappearance during a follow-up of a minimum of six months was considered. Likewise, the growth of an asymptomatic nail spicule was regarded as a post-operatory sequel and not as a recurrence
Muriel-Sánchez (2021) [51]	The growth of asymptomatic nail spicule was considered a sequel and not a recurrence
Peyvandi (2011) [52]	No definition provided
Shaath (2005) [53]	No definition provided
Tatlican (2009) [20]	Recurrence was defined as the formation of a new nail particule and the presence of any sign related with the re-ingrowth of the operated nail such as pain, erythema or spicule formation
Uygur (2016) [54]	No definition applied at the study start; applied definition to see the impact on study results “Had recurrence been defined as a need for repeat surgery, the recurrence rate of the group treated using our new technique would be zero”
Van der Ham (1990) [55]	No definition provided
Varma (1983) [26]	Symptomatic recurrence was defined as recurrence of a nail spike associated with persistent discomfort, pain and/or inflammation over a period of at least 8 weeks, for which the patient opted to have another operation
Wallace (1979) [24]	Unclear in their reporting of recurrence recording the ‘number of successes/number of failures’

##### Chemical matrixectomy vs conservative management

**Fig. 2:**
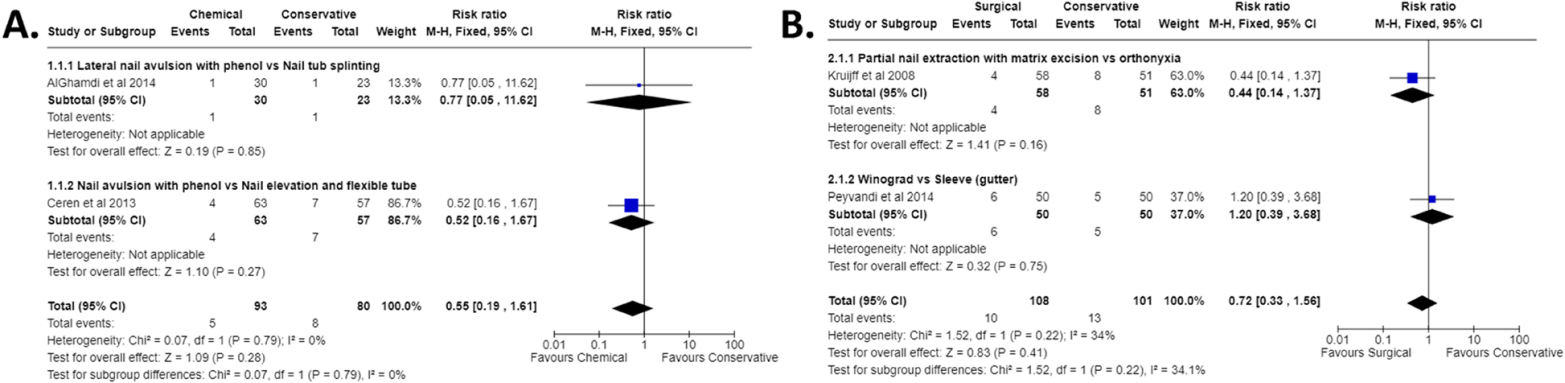
Meta-analysis comparing **A** Chemical matrixectomy vs Conservative management and **B** Surgical matrixectomy vs Conservative management

Two studies [29, 36] found that phenol matrixectomy did not significantly decrease the rate of recurrence when compared to a conservative approach such as nail tube splinting or nail elevation and flexible tubing (RR 0.55 [95% CI 0.19 to 1.61], I^2^ 0%; *p* = 0.280) (Fig. 2).

##### Surgical matrixectomy vs conservative management

Two studies [46, 52] compared surgical matrixectomy to a conservative approach, however neither method was significantly more effective at preventing recurrence (RR 0.72 [95% CI 0.33 to 1.56] I^2^ 34%; *p* = 0.410) (Fig. 2). Conversely, one study [24] that could not be included in this meta-analysis reported the wedge resection (27/32, 84%) to be superior to the gutter treatment (20/36, 56%) in terms of ‘number of successes’ (*p* < 0.05).

##### Chemical matrixectomy vs surgical matrixectomy

**Fig. 3:**
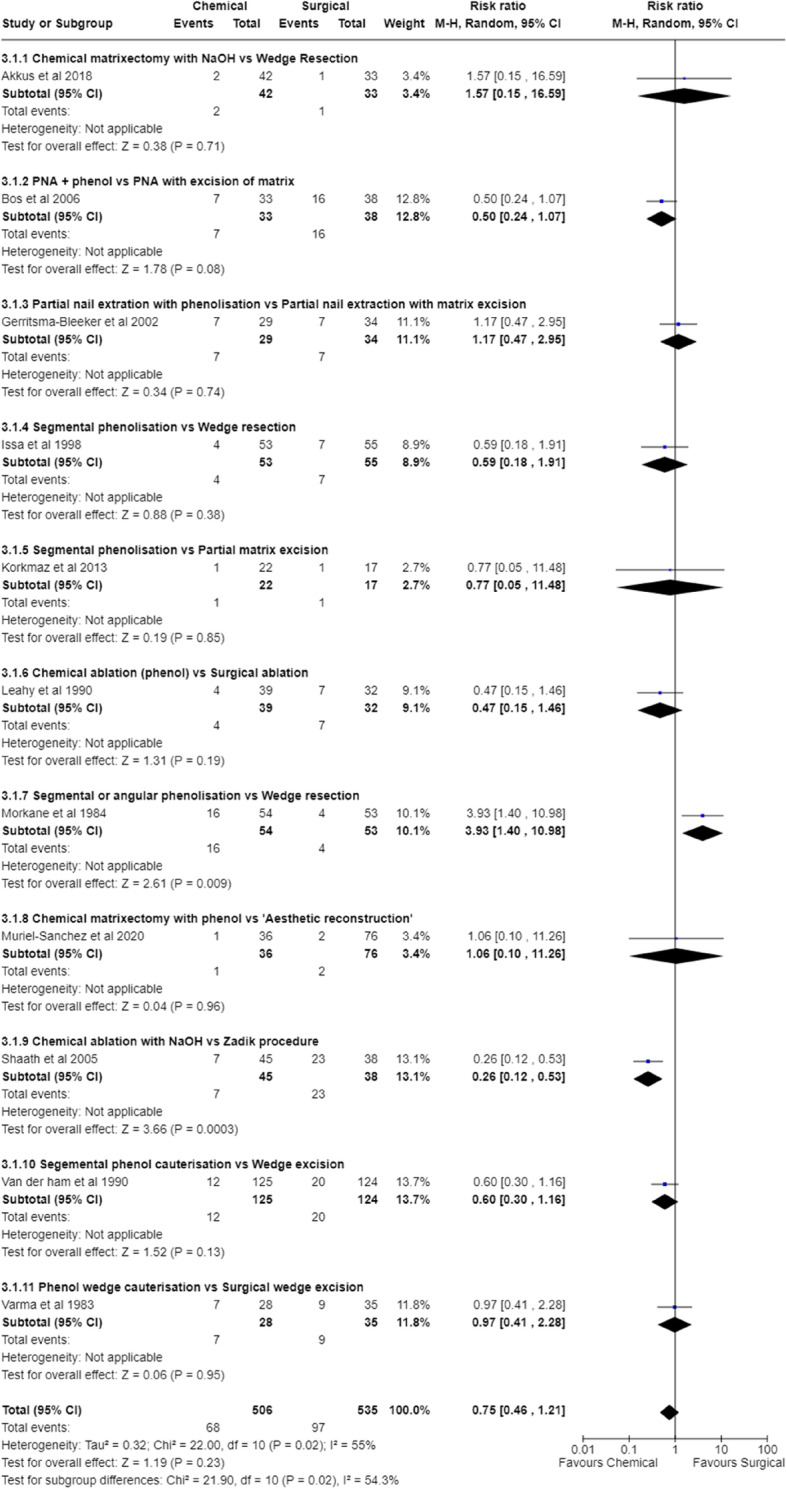
Meta-analysis comparing Chemical matrixectomy vs Surgical matrixectomy

Combining the eleven studies [26, 28, 35, 38, 41, 45, 47, 49, 50, 53, 55] where chemical matrixectomy was compared to surgical matrixectomy, found no significant difference in their ability to prevent recurrence (RR 0.75 [95% CI 0.46 to 1.21] I^2^ 55%; *p* = 0.230) (Fig. 3). In addition, funnel plots suggest an absence of publication bias (Supplementary File 2) as these data are symmetrically distributed.

##### Chemical matrixectomy vs chemical matrixectomy

Two studies [27, 33] compared phenol to trichloroacetic acid, however neither chemical proved to be more effective at preventing recurrence (RR 0.19 [95% CI 0.01 to 3.80] *p* = 0.280) (Supplementary Fig. 1).

##### Surgical matrixectomy vs other (e.g., CO_2_ laser, electrocautery)

In three studies [34, 42, 44], a surgical intervention (partial nail matrixectomy or curettage) did not significantly decrease the rate of recurrence when compared to an alternative method of matrixectomy such as electrocautery or CO_2_ laser (RR 1.61 [95% CI 0.88 to 2.95] I^2^ 37%; *p* = 0.120) (Supplementary Fig. 2).

##### Chemical matrixectomy vs other (e.g., CO_2_ laser, electrocautery)

Similarly, when comparing chemical matrixectomy to an alternative method of matrixectomy in two studies [25, 48], there was no significant difference in prevention of recurrence (RR 0.58 [95% CI 0.25 to 1.37] I^2^ 0%; *p* = 0.220) (Supplementary Fig. 3).

##### Avulsion vs avulsion + chemical matrixectomy

Avulsion with phenol matrixectomy was compared with nail avulsion alone in two studies [39, 43]. There was a significant reduction of recurrence in favour of phenol matrixectomy (RR 0.13 [95% CI 0.06 to 0.27] I^2^ 0%; *p* < 0.001) (Supplementary Fig. 4). Although Greig and colleagues [39] called their procedure ‘Nail edge excision’ the procedure described was the same as partial avulsion.

##### Surgical matrixectomy vs surgical matrixectomy

One study [54], compared the Winograd procedure using a new suturing technique, compared to the same surgical intervention and a traditional suturing technique. After 12 months, participants were asked to report any recurrence via telephone. The new suturing technique was more effective at preventing recurrence compared to the traditional technique (RR 0.42 [95% CI 0.21 to 0.85]) (Supplementary Fig. 5).

Another compared central toenail resection to wedge toenail resection [40]. After 6 months, the central toenail resection was considered more effective at preventing recurrence compared with the wedge toenail resection (RR 0.05 [95% CI 0.0 to 0.79]) (Supplementary Fig. 6).

##### Surgical matrixectomy vs surgical + chemical matrixectomy

Two studies compared a surgical intervention, either nail bed excision or wedge resection, with the same surgical intervention plus the addition of phenol [32, 41]. However, addition of phenol was not significantly more effective at preventing recurrence (RR 3.68 [95% CI 0.20 to 67.35] I^2^ 76%; *p* = 0.380) (Supplementary Fig. 7).

##### Chemical matrixectomy vs surgical + chemical matrixectomy

In two studies [31, 41], surgical matrixectomy plus phenolisation did not significantly decrease the rate of recurrence when compared to phenolisation alone (RR 1.92 [95% CI 0.06 to 62.30] I^2^ 62%; *p* = 0.710) (Supplementary Fig. 8).

##### Local anaesthetic vs local anaesthetic + adrenaline (epinephrine)

Two studies [30, 37] compared local anaesthetic (4 mL solution of 2% mepivacaine; 2% lidocaine, respectively), with a combination of the same local anaesthetic plus adrenaline (epinephrine). The use of adrenaline did not significantly decrease the rate of recurrence (RR 1.03 [95% CI 0.22 to 4.86] I^2^ 0%; *p* = 0.970) (Supplementary Fig. 9).

##### Chemical application time: 30 vs 60 s

Four studies used the same surgical intervention but varied the duration that the chemical was applied during the matrixectomy [20, 22, 23, 51]. Of these, three studies [20, 22, 23] could not be included in the meta-analysis, and none reported significant differences in chemical timing applications. The study by Gem and colleagues [22] compared chemical ablation with either 3 min of 80% phenol or 2 min of 10% sodium hydroxide and the second [23] compared either 1 or 2 min with 10% sodium hydroxide. Of the 422 procedures, 148 were lost to follow up, leaving 248/274 (study 1 *n* = 140/157; study 2 *n* = 108/118) who were completely asymptomatic at 18 months. No significant differences were found between the interventions. The numerical data was reproduced faithfully from the publication. There is an arithmetical error, but this has not been corrected due to uncertainty where it occurs. Lastly, Tatlican and colleagues [20] compared phenol with partial nail avulsion at 1, 2 and 3 min on rates of recurrence, assessed every 6 months over 24 months, and found no significant difference between the three groups (*p* = 0.092).

Of the one study [51] that was included in the meta-analysis, Muriel-Sanchez and colleagues compared the recurrence rate between phenol applications of 30 or 60 s, finding the 60-s application was more effective at preventing recurrence compared to the 30-s phenol application (RR 2.00 [95% CI 0.19 to 21.41]) (Supplementary Fig. 10).

##### Antibiotics vs no antibiotics

Bos and colleagues explored the use of topical antibiotics (5.3 mg soluble tablet of gentamicin applied locally) on recurrence, with and without matrix excision and phenol [35]. After 12 months the use of topical antibiotics alongside a chemical or surgical matrixectomy did not significantly decrease the rate of recurrence (RR 0.54 [95% CI 0.12 to 2.52] I^2^ 58%; *p* = 0.430) (Supplementary Fig. 11).

#### Relief of symptoms

Five studies assessed relief of symptoms [22, 23, 38, 40, 46]. Two studies assessed symptoms using a visual analogue scale ranging from 0 to 10 [38, 46], the remaining three studies did not specify the instrument used [22, 23, 40]. No definitions were provided for relief of symptoms.

##### Chemical procedures

In two studies [22, 23], no statistically significant differences were identified between patients receiving 3 min application of 80% phenol, 2 min of 10% sodium hydroxide and 1 min of 10% sodium hydroxide. However, Gem and colleagues did report 91% of all participants were asymptomatic after a minimum follow up time of 12 months (study 1) [22] and 18 months (study 2) [23].

##### Chemical and surgical procedures

Despite a tendency in the matrix group to have fewer persisting symptoms, the study by Gerritsma-Bleeker and colleagues [38] found no significant differences between partial nail extraction with phenolisation and partial nail extraction with matrix excision at 1, 3 or 12 months (*p* = 0.130, *p* = 0.270, *p* = 0.290, respectively).

##### Surgical procedures

Habeeb and colleagues [40] showed central toenail resection was significantly better in relieving symptoms compared to wedge toenail resection after 4 and 8 weeks (both *p* = 0.001).

##### Surgical and conservative procedures

Following receipt of either partial nail extraction with partial matrix excision or orthonyxia, no differences were noted in Kruijff and colleagues [46] study after 12 months.

### Ongoing studies

One ongoing clinical trial (CTRI/2017/09/009951) of interest was identified. Registered in 2017, this study remains classified as ‘Not yet recruiting’. Attempts were made to obtain an update on progress from the listed investigators but with no success. Three trial registries of interest were also identified (NCT03732313; IRCT201604176403N6; ACTRN12619001719123), however results were already included in this review [40, 42, 51].

### Risk of bias

**Fig. 4:**
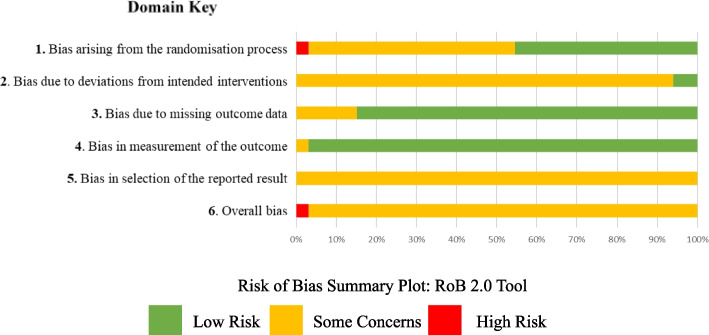
Risk of Bias Summary Plot: RoB 2.0 Tool

We used the used the Cochrane RoB 2.0 tool and assessed six domains for each study. No study was rated as low risk, for reasons such as not or providing information surrounding the randomisation process, not including all participants in the final analysis and failing to provide information on blinding of participants or the outcome assessor. Risk of bias summaries are presented in Fig. 4 and risk of bias table in Supplementary Table 2.

### Certainty of evidence

The certainty of evidence (Supplementary Table 3) for the outcome recurrence was: very low for meta-analyses comparing surgical vs conservative (2 RCTs, *n* = 209), chemical vs surgical (11 RCTs, *n* = 1041), surgical vs other (3 RCTs, *n* = 388), chemical vs avulsion (2 RCTs, *n* = 263), surgical vs surgical + chemical (2 RCTs, *n* = 171), chemical vs other (2 RCTs, *n* = 160), chemical vs surgical + chemical (2 RCTs, *n* = 191), epinephrine vs without epinephrine (2 RCTs, *n* = 114). Low for chemical vs conservative (2 RCTs, *n* = 173), phenol vs trichloroacetic acid (2 RCTs, *n* = 187) and surgical vs surgical + suturing (1 RCT, *n* = 128). Moderate for surgical vs surgical (1 RCT, *n* = 100), chemical timings (1 RCT, *n* = 108) and antibiotics (1 RCT, *n* = 117). The main reasons for downgrading the evidence were risk of bias, indirectness of evidence and imprecision.

## Discussion

Ingrown toenails, or onychocryptosis is a common nail pathology. Surgical resolution is often sought when conservative measures fail, or even as a first line intervention in more severe cases. As such, nail surgery is one of the most commonly performed procedures by podiatrists in the UK [11]. Despite the high number of publications on the topic, there has been a lack of robust systematic reviews covering the spectrum of surgical options in the decade since the last Cochrane review [4].

This review followed Cochrane methodology and conducted a prospectively registered systematic review with meta-analysis of surgical treatments for ingrown toenails. This paper includes a detailed description of our methodology and presents findings from our predefined primary outcomes: recurrence and relief of symptoms. Analysis of secondary outcomes will follow in a subsequent publication.

The systematic, search identified 1,641 potential publications which, after screening, enabled 36 studies with 3,756 participants covering a range of techniques that were included in the review. This is a substantial increase on the 24 and 18 studies in the previous Cochrane review [4], and the review by Vinay and colleagues [13], respectively. Recurrence was reported in all but one study, although there were variations in how this was defined and captured. Meta-analysis did not demonstrate a difference in risk of regrowth for most comparisons. Perhaps unsurprisingly, phenolisation was a notable exception to this pattern and when compared to nail avulsion alone, there was a very low certainty of evidence that use of phenol significantly reduced the risk of recurrence [39, 43]. However, use of phenol combined with surgical excision offered no benefit over phenolisation alone [31, 41]. In terms of how long to apply the phenol for, there was a moderate certainty of evidence that application of 1 min had lower risk of regrowth compared to 30 s [51], but there was no additional benefit when it was applied for 2 or 3 min [20]. Studies of peri-operative factors beyond the actual procedure such as use of different local anaesthetic with / without adrenaline [30, 37] and topical antibiotics [35] did not affect rates of regrowth even with such an atypical application technique.

Surprisingly, symptom relief was only reported in five [22, 23, 38, 40, 46] of the 36 studies and in three of those, it was not clear whether this was patient reported, or determined by the clinicians [22, 23, 40]. Even in these studies, exactly what ‘symptoms’ refers to is often unclear. Ingrown toenails are intensely painful though, and that this is rarely captured is a poor reflection on the quality of research in the field: it is no longer acceptable for studies to fail to capture key outcomes that matter to patients, and instead only focus on clinician reported outcomes. The importance of PROMs is well recognised by major national health policy and regulatory authorities [56, 57]. The authors question whether it is acceptable for future trials in ingrown toenails to continue to omit patient reported outcomes. It is important that future clinical trials differentiate between regrowth, which may be asymptomatic, and regrowth which causes pain and infection.

Clinical conclusions from this paper should be interpreted in line with our second paper that considers the secondary outcomes from our review: healing time, postoperative complications, pain of operation, postoperative pain (duration and intensity), and participant satisfaction. Only with these can a broader, more holistic, assessment of outcome be fully appreciated so these are essential for guiding practice.

All 36 studies included in the review were assessed as being either high risk or having some concerns about bias when assessed with the Cochrane RoB 2.0 tool. Similarly, out of the 15 comparisons made here, most were considered to have either very low, or low certainty of evidence when assessed with the GRADE system. Only three reached moderate, and none were considered to have high certainty. The main reasons for downgrading the evidence were risk of bias, indirectness of evidence, and imprecision. In addition, surgical technique was often poorly described, and there was large variation in the use of terms such as recurrence. It is also important that future clinical trials differentiate between regrowth that may be asymptomatic and regrowth that causes pain and infection. To put this another way, 3,756 people have taken part in research studies that do little to guide clinical practice. Some of this may be due to poor reporting, but poor design also plays a major role. Regardless, both of these reasons can, and should be avoided and this topic has been widely discussed in the literature with recommendations made to improve research across healthcare [58–60]. Findings from this review differ from those of previous reviews. In part, this may be explained by the publication of new research in the decade since the Cochrane review [4] and a broader focus than the review by Vinay and colleagues specific to phenol [13]. However, the risk of bias assessment was also different as was the grading of strength of recommendations that could be made. Whilst there would inevitably be some variation in these relatively subjective assessment systems the authors stand by this assessment and have discussed some of the methodological limitations in the existing evidence base that have led us to this conclusion. More, high quality clinical trials to inform clinical decision making are urgently needed in nail surgery.

This review and meta-analysis both have strengths and limitations. The authors consider the robust methodology of the search, screening, extracted data, synthesis, meta-analysis, and use of tools such as RoB 2.0 and GRADE as methodological strengths. Deliberate attempts have been made to ensure that comparisons within the meta-analysis are clinically meaningful. Whilst some readers may disagree with how these studies have been compared, or want additional comparisons, they have been made in an open and transparent way. As a further note, this process was made more difficult due to the poor procedure descriptions, with many describing more than one procedure i.e., stated as nail edge excision but partial avulsion was described. Well established reporting guidelines such as the SUPER and IDEAL frameworks should be followed in the future [61, 62].

## Conclusion

This paper presents the co-primary outcomes from a systematic review with meta-analysis that should be interpreted in conjunction with its second paper. Despite the high number of publications on this topic, the quality of research was poor and the conclusions that can be inferred from existing trials is limited. Phenolisation of the nail matrix reduces the risk of recurrence following nail ablation, and 1 min appears to be the optimum time for application but there is less certainty around this recommendation. Further research is needed to explore the effectiveness of other commonly used ablative agents such as sodium hydroxide and to systematically explore the optimisation of post-operative care.

## Supplementary Information

**Additional file 1: Supplementary Figure 1.** Chemical vs Chemical. Forest plot of risk of recurrence for chemical matrixectomy with phenol compared to matrixectomy with trichloroacetic acid.

**Additional file 2: Supplementary Figure 2.** Surgical vs Other. Forrest plot of risk of recurrence for surgical matrixectomy compared to matrixectomy by other techniques.

**Additional file 3: Supplementary Figure 3.** Chemical vs Other. Forrest plot of risk of recurrence for chemical matrixectomy with pheonol compared to electrocautery.

**Additional file 4: Supplementary Figure 4.** Avulsion vs Avulsion + Chemical. Forrest plot of risk of recurrence for avulsion compared to avulsion plus chemical matrixextomy with phenol.

**Additional file 5: Supplementary Figure 5.** Surgical vs Surgical. Forrest plot of risk of recurrence for Winograd Procedure involving a new suturing technique compared to Winograd Procedure plus traditional suturing technique.

**Additional file 6: Supplementary Figure 6.** Surgical vs Surgical. Forrest plot of risk of recurrence for Central toenail resection compared to Wedge toenail resection.

**Additional file 7: Supplementary Figure 7.** Surgical vs Surgical + Chemical. Forrest plot of risk of recurrence for surgical matrixectomy compared to surgical matrixectomy plus chemical ablation.

**Additional file 8: Supplementary Figure 8.** Chemical vs Surgical + chemical. Forrest plot of risk of recurrence for chemical matrixectomy compared to surgical procedure plus chemical matrixectomy.

**Additional file 9: Supplementary Figure 9.** Anaesthetic vs Anaesthetic + Adrenaline. Forrest plot of risk of recurrence for local anaesthetic compared to local anaesthetic plus adrenaline.

**Additional file 10: Supplementary Figure 10.** Chemical timings. Forrest plot of risk of recurrence for 30 second application of phenol compared to 60 second application of phenol.

**Additional file 11: Supplementary Figure 11.** Antibiotics vs No Antibiotics. Forrest plot of risk of recurrence for procedure with antibiotic compared to procedure without antibiotic.

**Additional file 12: Supplementary File 1.** Full Search Strategy.

**Additional file 13: Supplementary File 2.** Funnel Plot: Recurrence.

**Additional file 14: Supplementary Table 1.** Table of Excluded Studies.

**Additional file 15: Supplementary Table 2.** Risk of Bias Summary Table.

**Additional file 16: Supplementary Table 3.** Certainty of evidence using the GRADE approach.

## Data Availability

All data are available from the corresponding author on reasonable request.

## Abbreviations

CENTRAL: Cochrane Central Register of Controlled Trials
CI: Confidence Interval
GRADE: Grades of Research, Assessment, Development and Evaluation
ISRCTN: International Clinical Trials Registry
MD: Mean Differences
MESH: Medical Subject Heading
PRISMA: Preferred Reporting Items for Systematic Reviews and Meta-Analyses
PROMs: Patient Reported Outcome Measures
RCT: Randomised Controlled Trial
ROB: Risk of Bias
RR: Risk Ratio
