# Supplementary figures and images for: A systematic review and meta-analysis of randomised controlled trials on surgical treatments for ingrown toenails part I: recurrence and relief of symptoms
Source: J Foot Ankle Res. 2023 Jun 10;16:35. doi: 10.1186/s13047-023-00631-1 (PMC10257290; doi:10.1186/s13047-023-00631-1)

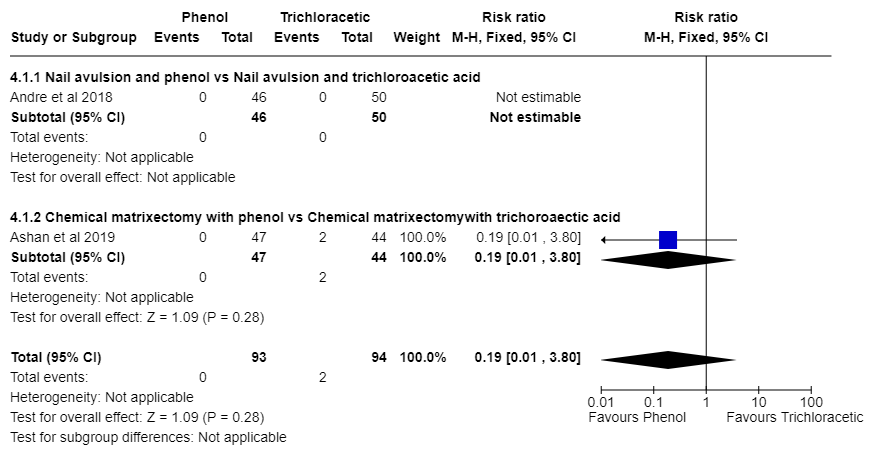

Supplement: Supplementary file 1 — Additional file 1: Supplementary Figure 1. Chemical vs Chemical. Forest plot of risk of recurrence for chemical matrixectomy with phenol compared to matrixectomy with trichloroacetic acid. [file 13047_2023_631_MOESM1_ESM.png]

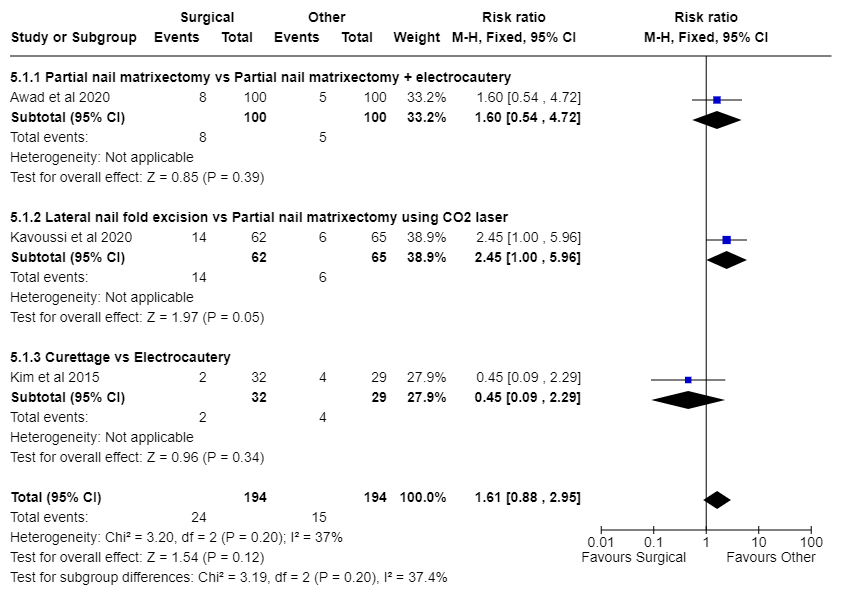

Supplement: Supplementary file 2 — Additional file 2: Supplementary Figure 2. Surgical vs Other. Forrest plot of risk of recurrence for surgical matrixectomy compared to matrixectomy by other techniques. [file 13047_2023_631_MOESM2_ESM.png]

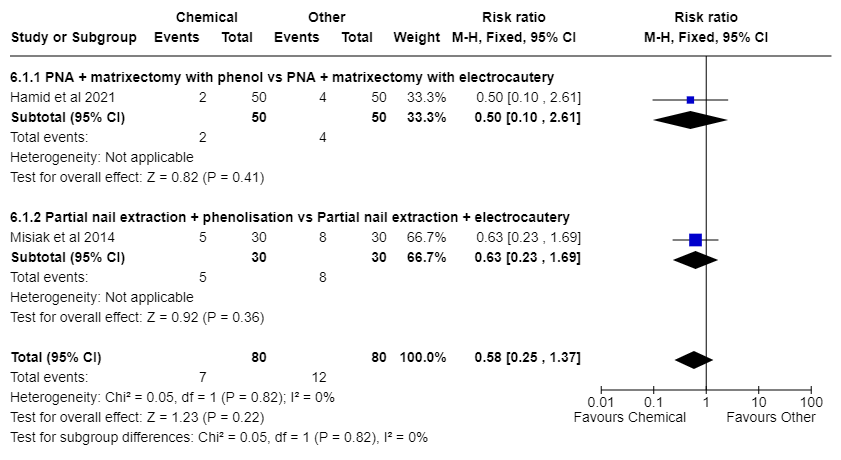

Supplement: Supplementary file 3 — Additional file 3: Supplementary Figure 3. Chemical vs Other. Forrest plot of risk of recurrence for chemical matrixectomy with pheonol compared to electrocautery. [file 13047_2023_631_MOESM3_ESM.png]

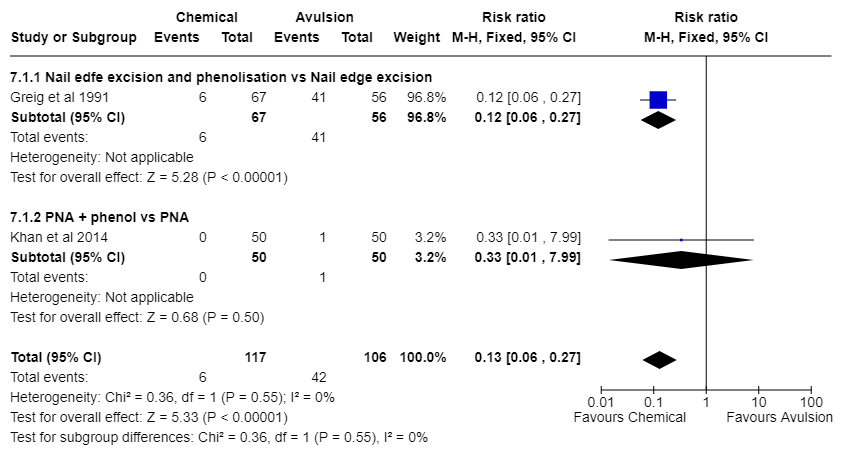

Supplement: Supplementary file 4 — Additional file 4: Supplementary Figure 4. Avulsion vs Avulsion + Chemical. Forrest plot of risk of recurrence for avulsion compared to avulsion plus chemical matrixextomy with phenol. [file 13047_2023_631_MOESM4_ESM.png]

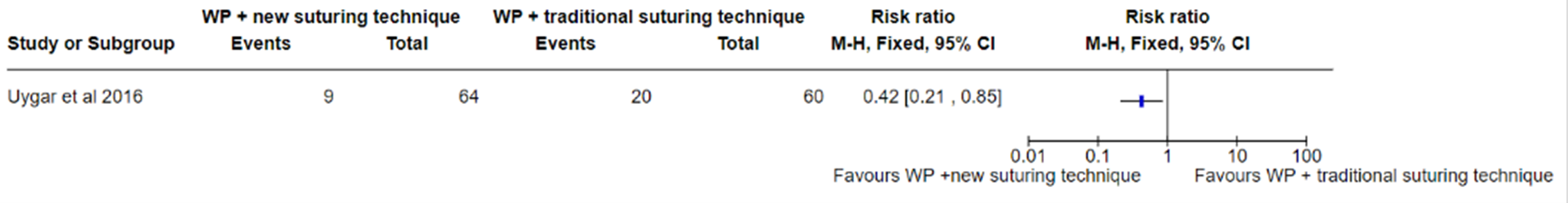

Supplement: Supplementary file 5 — Additional file 5: Supplementary Figure 5. Surgical vs Surgical. Forrest plot of risk of recurrence for Winograd Procedure involving a new suturing technique compared to Winograd Procedure plus traditional suturing technique. [file 13047_2023_631_MOESM5_ESM.png]

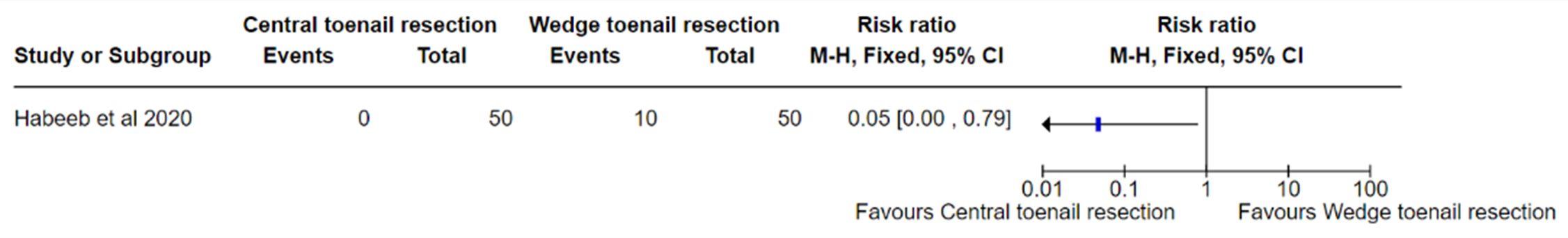

Supplement: Supplementary file 6 — Additional file 6: Supplementary Figure 6. Surgical vs Surgical. Forrest plot of risk of recurrence for Central toenail resection compared to Wedge toenail resection. [file 13047_2023_631_MOESM6_ESM.png]

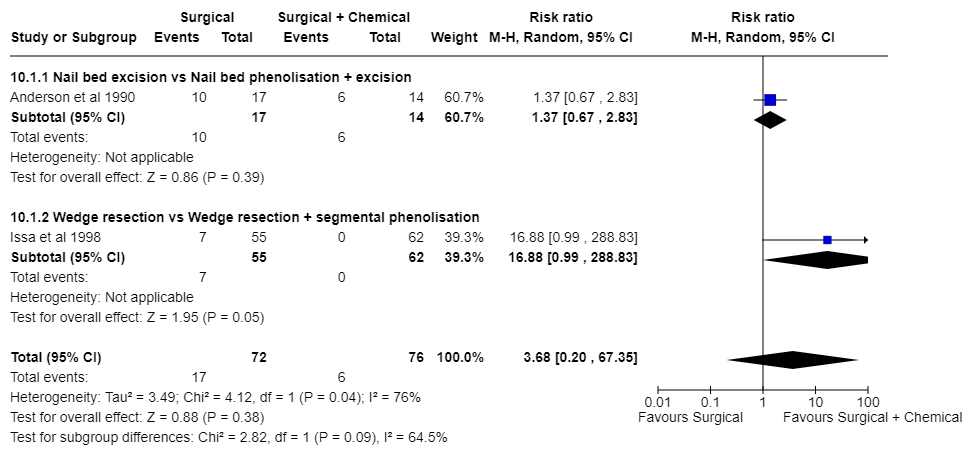

Supplement: Supplementary file 7 — Additional file 7: Supplementary Figure 7. Surgical vs Surgical + Chemical. Forrest plot of risk of recurrence for surgical matrixectomy compared to surgical matrixectomy plus chemical ablation. [file 13047_2023_631_MOESM7_ESM.png]

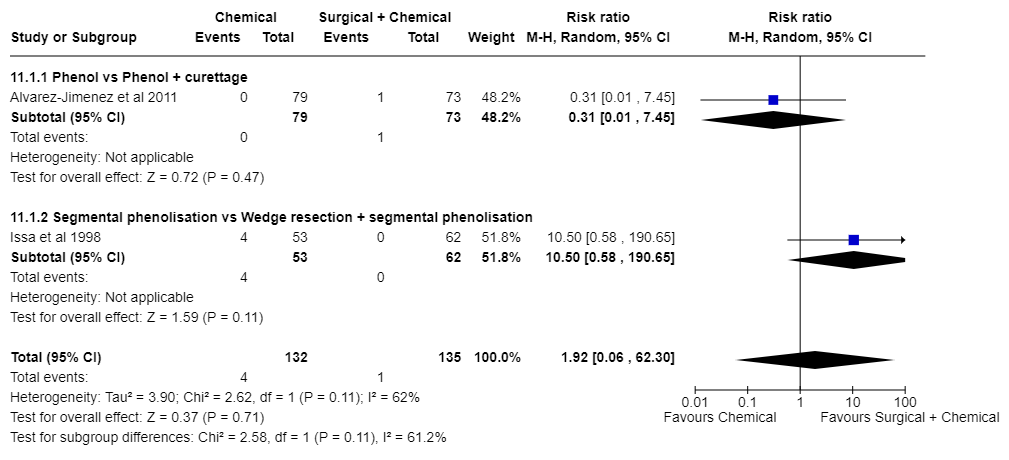

Supplement: Supplementary file 8 — Additional file 8: Supplementary Figure 8. Chemical vs Surgical + chemical. Forrest plot of risk of recurrence for chemical matrixectomy compared to surgical procedure plus chemical matrixectomy. [file 13047_2023_631_MOESM8_ESM.png]

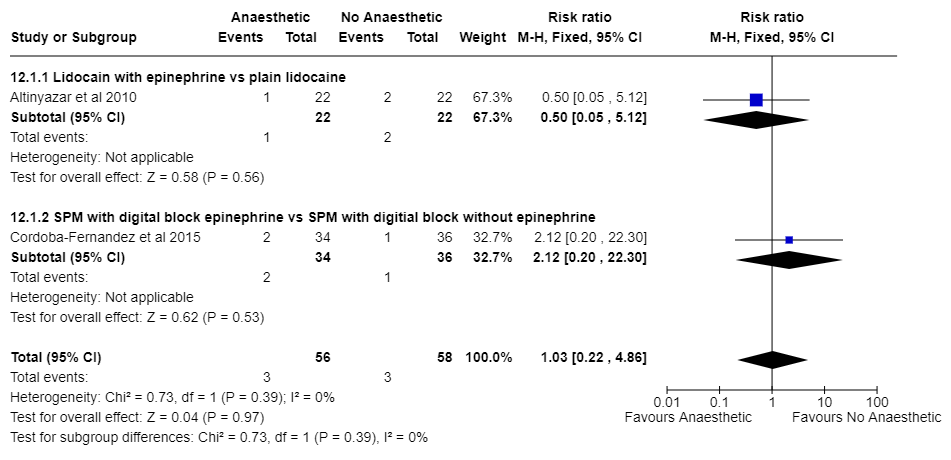

Supplement: Supplementary file 9 — Additional file 9: Supplementary Figure 9. Anaesthetic vs Anaesthetic + Adrenaline. Forrest plot of risk of recurrence for local anaesthetic compared to local anaesthetic plus adrenaline. [file 13047_2023_631_MOESM9_ESM.png]

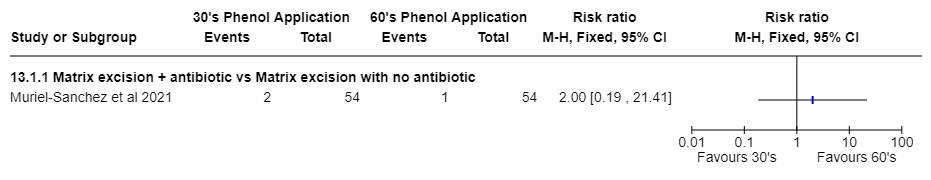

Supplement: Supplementary file 10 — Additional file 10: Supplementary Figure 10. Chemical timings. Forrest plot of risk of recurrence for 30 second application of phenol compared to 60 second application of phenol. [file 13047_2023_631_MOESM10_ESM.png]

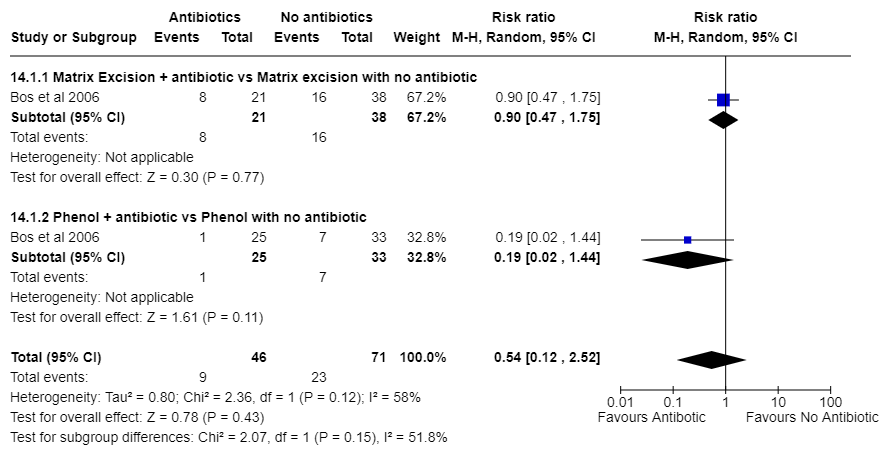

Supplement: Supplementary file 11 — Additional file 11: Supplementary Figure 11. Antibiotics vs No Antibiotics. Forrest plot of risk of recurrence for procedure with antibiotic compared to procedure without antibiotic. [file 13047_2023_631_MOESM11_ESM.png]
