# Supplementary material for: A systematic review and meta-analysis of randomised controlled trials on surgical treatments for ingrown toenails part I: recurrence and relief of symptoms
Source: J Foot Ankle Res. 2023 Jun 10;16:35. doi: 10.1186/s13047-023-00631-1 (PMC10257290; doi:10.1186/s13047-023-00631-1)
Supplement: Supplementary file 12 — Additional file 12: Supplementary File 1. Full Search Strategy. [file 13047_2023_631_MOESM12_ESM.docx]

**Supplementary File 1.** Full Search Strategy

Ingrow* nail*

ingrow* toenail*

onychocryptosis

unguis incarnatus

embedded nail*

embedded toenail*

involuted nail*

involuted toenail*
