# Supplementary material for: A systematic review and meta-analysis of randomised controlled trials on surgical treatments for ingrown toenails part I: recurrence and relief of symptoms
Source: J Foot Ankle Res. 2023 Jun 10;16:35. doi: 10.1186/s13047-023-00631-1 (PMC10257290; doi:10.1186/s13047-023-00631-1)
Supplement: Supplementary file 13 — Additional file 13: Supplementary File 2. Funnel Plot: Recurrence. [file 13047_2023_631_MOESM13_ESM.docx]

**Supplementary File 2.** Funnel Plot: Recurrence


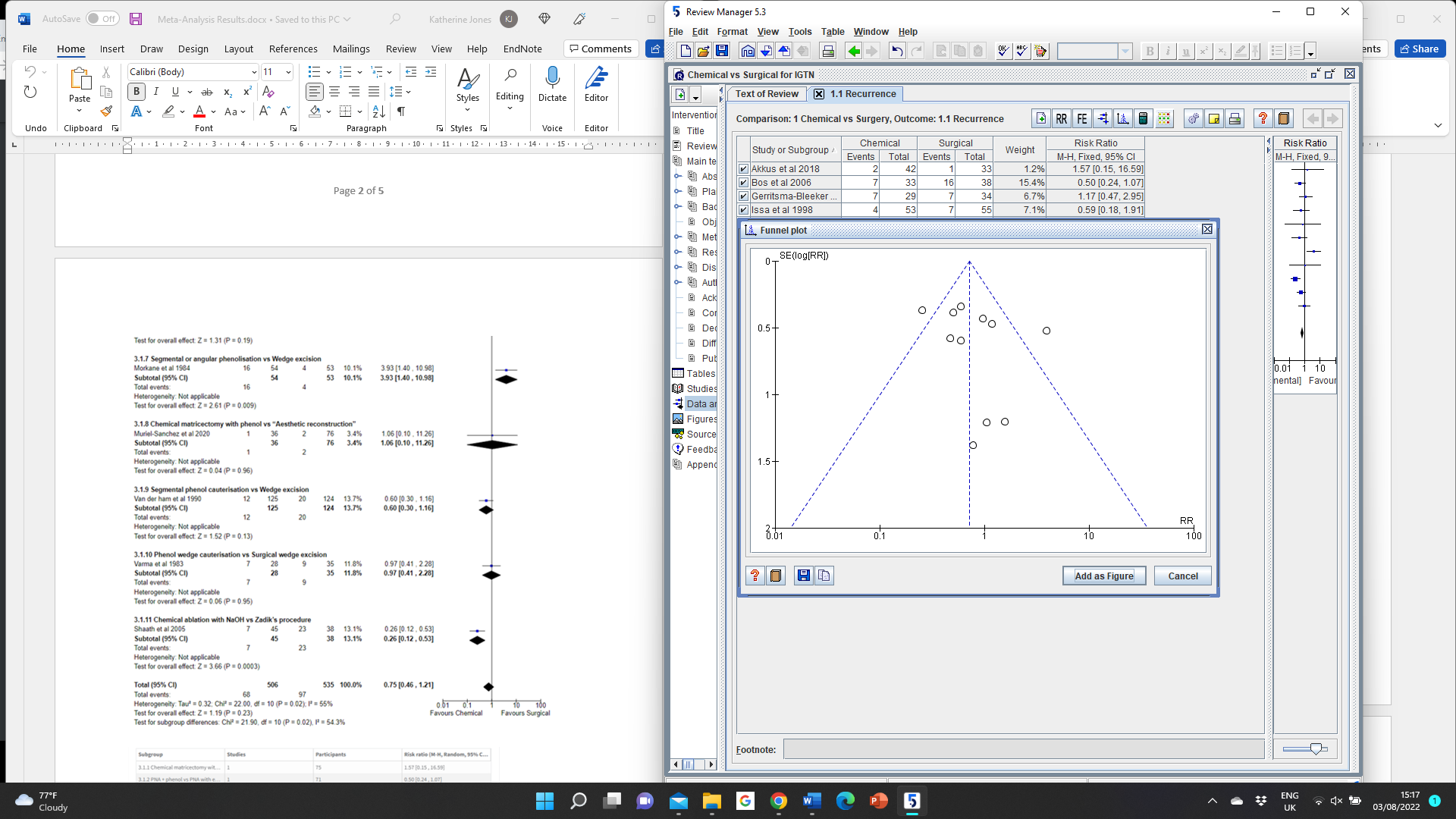


Funnel plot of comparison: chemical vs surgical for recurrence. SE, Standard Error; RR, Risk Ratio
