# Supplementary material for: A systematic review and meta-analysis of randomised controlled trials on surgical treatments for ingrown toenails part I: recurrence and relief of symptoms
Source: J Foot Ankle Res. 2023 Jun 10;16:35. doi: 10.1186/s13047-023-00631-1 (PMC10257290; doi:10.1186/s13047-023-00631-1)
Supplement: Supplementary file 14 — Additional file 14: Supplementary Table 1. Table of Excluded Studies. [file 13047_2023_631_MOESM14_ESM.docx]

**Supplementary Table 1.** Table of Excluded Studies

| **Author** | **Study Title** | **Reason for Exclusion** |
| --- | --- | --- |
| ACTRN12619001719123 | Cauterization of the germinal nail matrix using phenol applications of 30 or 60": a randomized double-blind clinical trial | Registration. Study (Muriel-Sánchez et al., 2021) included |
| Aksakal | Minimizing postoperative drainage with 20% ferric chloride after chemical matricectomy with phenol | Not an RCT |
| Arista | Onychocriptosis: a comparative study of postoperatory lateral partial matricectomy vs lateral partial matricectomy with phenol | Not in English |
| Andrew | Nail bed ablation--excise or cauterise? A controlled study | Quasi randomised |
| Becerro | Efficacy of Preoperative and Intraoperative Skin and Nail Surgical Preparation of the Foot in Reducing Bacterial Load | Wrong outcomes |
| Blake | A post-operative comparison of nail avulsion using phenol and cryotherapy. | Wrong study design |
| Bostanci | Comparison of phenol and sodium hydroxide chemical matricectomies for the treatment of ingrowing toenails | Quasi randomised |
| Bostanci | Chemical Matricectomy With Sodium Hydroxide: Long-Term Follow-up Results | Wrong study design |
| Burssens | A comparative study of 2 treatment methods for onychocryptosis (ingrown toenail) | Quasi randomised |
| Cameron | Ingrowing toenails: an evaluation of two treatments | Not an RCT |
| Cologlu | A new anatomical repair method for the treatment of ingrown nail: prospective comparison of wedge resection of the matrix and partial matricectomy followed by lateral fold advancement flap. | Not an RCT |
| CTRI/2017/09/009951 | Comparing two methods of treatment of ingrown toe nails: removing part of the nail and using phenol to destroy the nail origin versus removing part of the nail and surgically removing the nail origin | Registration |
| Flores | Partial matricectomy vs partial matricectomy with electrofulguration as a treatment for onychocryptosis | Not in English |
| Grover | Controlled trial comparing the efficacy of 88% phenol versus 10% sodium hydroxide for chemical matricectomy in the management of ingrown toenail. | Quasi randomised |
| Harrer | Treatment of ingrown toenails using a new conservative method - a prospective study comparing brace treatment with Emmerts procedure | Quasi randomised |
| Herold | A prospective comparison of wedge matrix resection with nail matrix phenolization for the treatment of ingrown toenail. | Quasi randomised |
| Ince | Comparison between knot and Winograd techniques on ingrown nail treatment | Quasi randomised |
| IRCT201604176403N6 | Treatment of ingrowing nail by CO2 laser and lateral nail fold excision | Registration. Study (Kavoussi et al., 2020) already included |
| Isik | Comparison of partial matrixectomy and combination treatment (partial matrixectomy + phenol) in ingrown toenail. | Wrong study design |
| Kim | Ultrasound-guided popliteal nerve block with short-acting lidocaine in the surgical treatment of ingrown toenails | Not an RCT |
| Kocygit | Sodium hydroxide chemical matricectomy for the treatment of ingrown toenails: Comparison of three different application periods | Quasi randomised |
| Kucuktas | Comparison of effectiveness of electrocautery and cryotherapy in partial matrixectomy after partial nail extraction in the treatment of ingrown nails | Not an RCT |
| Lin | A surgical approach to ingrown nail: Partial matricectomy using CO2 laser | Wrong study design |
| Mori | Ingrown nails: a comparison of the nail matrix phenolization method with the elevation of the nail bed-periosteal flap procedure. | Wrong study design |
| Ozan | Partial matricectomy with curettage and electrocautery: Acomparison of two surgical methods in the treatment of ingrown toenails | Not an RCT |
| Ozdemir | Chemical matricectomy with 10% sodium hydroxide for the treatment of ingrowing toenails | Not an RCT |
| Pamuk | Winograd surgery can be performed under partial digital block in patients with ingrown toenails | Not an RCT |
| Perez-Ray | Exploring postoperative outcomes for ingrown toenails. naoh vs wedge resection techniques | Wrong study design |
| Pettine | Ingrown toenail: results of surgical treatment. | Wrong study design |
| Ramesh | Comparative efficacy of 10% sodium hydroxide, 88% phenol, and 90% trichloroacetic acid as chemical cauterants for partial matricectomy in the management of great toe nail onychocryptosis | Not an RCT |
| Shaath | Economic implications and recurrencies favour chemical against surgical ablation for ingrowing toenails treatment | Abstract only; Not enough information |
| Tait | Surgical or phenol ablation of the nail bed for ingrowing toenails: a randomised controlled trial. | Quasi randomised |
| Tait | A RANDOMIZED CONTROLLED TRIAL OF SURGICAL OR PHENOL ABLATION OF THE NAILBED IN THE TREATMENT OF INGROWING TOENAILS | Wrong study design |
| Talwar | A study on the surgical treatment of ingrowing toenail with nail excision with chemical matricectomy versus nail excision alone | Not an RCT |
| RCT, Randomised Controlled Trial | | |
