# Supplementary material for: A systematic review and meta-analysis of randomised controlled trials on surgical treatments for ingrown toenails part I: recurrence and relief of symptoms
Source: J Foot Ankle Res. 2023 Jun 10;16:35. doi: 10.1186/s13047-023-00631-1 (PMC10257290; doi:10.1186/s13047-023-00631-1)
Supplement: Supplementary file 15 — Additional file 15: Supplementary Table 2. Risk of Bias Summary Table. [file 13047_2023_631_MOESM15_ESM.docx]

| **Supplementary Table 2.** Risk of Bias Summary Table | | | | | | |
| --- | --- | --- | --- | --- | --- | --- |
| **Study** | **Domain** | | | | | |
|  | **1** | **2** | **3** | **4** | **5** | **Overall** |
| Ahsan (2019) |  |  |  |  |  |  |
| Akkus (2018) |  |  |  |  |  |  |
| AlGhamdi (2014) |  |  |  |  |  |  |
| Altinyazar (2010) |  |  |  |  |  |  |
| Alvarez-Jimenez (2011) |  |  |  |  |  |  |
| Anderson (1990) |  |  |  |  |  |  |
| Andre (2018) |  |  |  |  |  |  |
| Awad (2020) |  |  |  |  |  |  |
| Bos (2006) |  |  |  |  |  |  |
| Ceren (2013) |  |  |  |  |  |  |
| Cordoba-Fernandez (2015) |  |  |  |  |  |  |
| Gem (1990) a |  |  |  |  |  |  |
| Gem (1990) b |  |  |  |  |  |  |
| Gerritsma-Bleeker (2002) |  |  |  |  |  |  |
| Greig (1991) |  |  |  |  |  |  |
| Habeeb (2020) |  |  |  |  |  |  |
| Hamid (2021) |  |  |  |  |  |  |
| Issa (1998) |  |  |  |  |  |  |
| Kavoussi (2020) |  |  |  |  |  |  |
| Khan (2014) |  |  |  |  |  |  |
| Kim (2015) |  |  |  |  |  |  |
| Korkmaz (2013) |  |  |  |  |  |  |
| Kruijff (2008) |  |  |  |  |  |  |
| Leahy (1990) |  |  |  |  |  |  |
| Misiak (2014) |  |  |  |  |  |  |
| Morkane (1984) |  |  |  |  |  |  |
| Muriel-Sánchez (2020) |  |  |  |  |  |  |
| Muriel-Sánchez (2021) |  |  |  |  |  |  |
| Peyvandi (2011) |  |  |  |  |  |  |
| Reyzelman (2000) |  |  |  |  |  |  |
| Shaath (2005) |  |  |  |  |  |  |
| Tatlican (2009) |  |  |  |  |  |  |
| Uygur (2016) |  |  |  |  |  |  |
| Van der Ham (1990) |  |  |  |  |  |  |
| Varma (1983) |  |  |  |  |  |  |
| Wallace (1979) |  |  |  |  |  |  |

**Domain Key**

1: Randomisation process; 2: Deviations from the intended interventions; 3: Missing outcome data; 4: Measurement of the outcome; 5: Selection of the reported result

Low risk of bias; Some risk of bias; High risk of bias
