# Supplementary material for: A systematic review and meta-analysis of randomised controlled trials on surgical treatments for ingrown toenails part I: recurrence and relief of symptoms
Source: J Foot Ankle Res. 2023 Jun 10;16:35. doi: 10.1186/s13047-023-00631-1 (PMC10257290; doi:10.1186/s13047-023-00631-1)
Supplement: Supplementary file 16 — Additional file 16: Supplementary Table 3. Certainty of evidence using the GRADE approach. [file 13047_2023_631_MOESM16_ESM.docx]

| **Supplementary Table 3.** Certainty of evidence using the GRADE approach | | | | | | | | |
| --- | --- | --- | --- | --- | --- | --- | --- | --- |
| Outcome | No. of Studies/ No of participants | Study Design Grade | Risk of bias/certainty  (Downgrade 1 if ≥50% of domains rated as ‘some concerns’ or ‘high’ on risk of bias on Cochrane risk-of-bias tool version 2) | Indirectness of evidence  (Downgrade 1 if significant differences in PICO factors that may result in change of outcome) | Heterogeneity or inconsistency of effect  (Potential downgrade if wide variance of point estimates across studies; minimal/no overlap of CIs; wide (<2) PI or significant heterogeneity tests) | Imprecision  (Does the 95% CI cross the line of no effect? If yes, Downgrade 1.  If no, Is the OIS† reached? If no, Downgrade 1) | Publication bias^*^ | Certainty of Evidence |
| Recurrence  (Chemical vs Conservative management) | 2 RCTs ^27, 28^ n= 173 | High | Some concerns or high risk >50% downgrade 1 | No important indirectness = no change | No important inconsistency = no change | 95% CI does not exclude 0 or OIS not met = downgrade 1 | No evidence of publication bias= no change | Low  (⊙⊙○○) |
| Recurrence  (Surgical matrixectomy vs Conservative management) | 2 RCTs ^26, 29^ n= 209 | High | Some concerns or high risk >50% downgrade 1 | Difference in timepoints collected = downgrade 1 | No important inconsistency = no change | 95% CI does not exclude 0 or OIS not met = downgrade 1 | No evidence of publication bias= no change | Very Low  (⊙○○○) |
| Recurrence  (Chemical matrixectomy vs Surgical matrixectomy) | 11 RCTs ^25, 30, 31, 32, 33, 34, 35, 36, 37, 38, 39^ n= 1041 | High | Some concerns or high risk >50% downgrade 1 | Difference in timepoints collected = downgrade 1 | No important inconsistency = no change | 95% CI does not exclude 0 or OIS not met = downgrade 1 | No evidence of publication bias; Funnel plot symmetrical= no change | Very Low  (⊙○○○) |
| Recurrence  (Chemical matrixectomy vs Chemical matrixectomy) | 2 RCTs ^45, 47^ n= 187 | High | Some concerns or high risk >50% downgrade 1 | No important indirectness = no change | No important inconsistency = no change | 95% CI does not exclude 0 or OIS not met = downgrade 1 | No evidence of publication bias= no change | Low  (⊙⊙○○) |
| Recurrence  (Surgical matrixectomy vs Other [e.g., CO2 laser, electrocautery]) | 3 RCTs ^49, 50, 51^ n= 388 | High | Some concerns or high risk >50% downgrade 1 | Difference in timepoints collected = downgrade 1 | No important inconsistency = no change | 95% CI does not exclude 0 or OIS not met = downgrade 1 | No evidence of publication bias= no change | Very Low  (⊙○○○) |
| Recurrence (Chemical matrixectomy vs Other [e.g., CO2 laser, electrocautery]) | 2 RCTs ^24, 52^ n= 160 | High | Some concerns or high risk >50% downgrade 1 | Difference in timepoints collected = downgrade 1 | No important inconsistency = no change | 95% CI does not exclude 0 or OIS not met = downgrade 1 | No evidence of publication bias= no change | Very Low  (⊙○○○) |
| Recurrence (Avulsion vs Avulsion + Chemical matrixectomy) | 2 RCTs ^41, 44^ n= 263 | High | Some concerns or high risk >50% downgrade 1 | Difference in timepoints collected = downgrade 1 | No important inconsistency = no change | 95% CI does not exclude 0 or OIS not met = downgrade 1 | No evidence of publication bias= no change | Very Low  (⊙○○○) |
| Recurrence (Surgical matrixectomy vs Surgical matrixectomy: Central TNR vs Wedge TNR) | 1 RCT ^43^ n= 100 | High | Low risk= no change | No important indirectness = no change | No important inconsistency = no change | 95% CI does not exclude 0 or OIS not met = downgrade 1 | No evidence of publication bias= no change | Moderate  (⊙⊙⊙○) |
| Recurrence (Surgical matrixectomy vs Surgical matrixectomy: WP + new suturing vs WP + traditional suturing) | 1 RCT ^46^  n= 128 | High | Some concerns or high risk >50% downgrade 1 | No important indirectness = no change | No important inconsistency = no change | 95% CI does not exclude 0 or OIS not met = downgrade 1 | No evidence of publication bias= no change | Low  (⊙⊙○○) |
| Recurrence (Surgical matrixectomy vs Surgical + Chemical matrixectomy) | 2 RCTs ^33, 44^ n= 171 | High | Some concerns or high risk >50% downgrade 1 | Difference in timepoints collected = downgrade 1 | No important inconsistency = no change | 95% CI does not exclude 0 or OIS not met = downgrade 1 | No evidence of publication bias= no change | Very Low  (⊙○○○) |
| Recurrence  (Chemical matrixectomy vs Surgical + Chemical matrixectomy) | 2 RCTs ^34, 42^ n= 191 | High | Some concerns or high risk >50% downgrade 1 | Difference in timepoints collected = downgrade 1 | No important inconsistency = no change | 95% CI does not exclude 0 or OIS not met = downgrade 1 | No evidence of publication bias= no change | Very Low  (⊙○○○) |
| Recurrence (Epinephrine vs Without Epinephrine) | 2 RCTs ^53, 54^  n= 114 | High | Some concerns or high risk >50% downgrade 1 | Difference in timepoints collected = downgrade 1 | No important inconsistency = no change | 95% CI does not exclude 0 or OIS not met = downgrade 1 | No evidence of publication bias= no change | Very Low  (⊙○○○) |
| Recurrence (Chemical timings 30’s vs 60’s) | 1 RCT ^48^ n= 108 | High | Low risk= no change | No important indirectness = no change | No important inconsistency = no change | 95% CI does not exclude 0 or OIS not met = downgrade 1 | No evidence of publication bias= no change | Moderate  (⊙⊙⊙○) |
| Recurrence (Antibiotics vs No Antibiotics) | 1 RCT ^31^ n= 117 | High | Low risk= no change | No important indirectness = no change | No important inconsistency = no change | 95% CI does not exclude 0 or OIS not met = downgrade 1 | No evidence of publication bias= no change | Moderate  (⊙⊙⊙○) |
| Abbreviations: GRADE, Grades of Research, Assessment, Development and Evaluation; RCT, Randomised Controlled Trial; PICO, Patient, Intervention, Comparison and Outcome; CI, Confidence Interval; PI, Prediction interval; OIS, Optimal Information Size; TNR, Toenail Resection  †Where there was no power calculation for the outcome, the OIS required was presumed to be met if n≥400^23^  * Publication bias decision was based around the following considerations: study design and study size ^23^ | | | | | | | | |
